# Requirement of brain interleukin33 for aquaporin4 expression in astrocytes and glymphatic drainage of abnormal tau

**DOI:** 10.1038/s41380-020-00992-0

**Published:** 2021-01-12

**Authors:** Jean Wu, Colin Carlock, Junbo Shim, Ines Moreno-Gonzalez, William Glass, April Ross, Tatiana Barichello, Joao Quevedo, Yahuan Lou

**Affiliations:** 1 Department of Diagnostic Sciences, School of Dentistry, University of Texas Health Science Center at Houston, Houston, TX 77054 USA; 2 Department of Neurology, McGovern School of Medicine, University of Texas Health Science Center at Houston, Houston, TX 77030 USA; 3 Mitchell Center for Alzheimer’s Disease and Related Brain Disorders, McGovern School of Medicine, University of Texas Health Science Center at Houston, Houston, TX 77030 USA; 4 Networking Research Center on Neurodegenerative Diseases (CIBERNED), Department of Cell Biology, Facultad Ciencias, Universidad de Malaga, Malaga, Spain; 5 Department of Pathology, McGovern School of Medicine, University of Texas Health Science Center at Houston, Houston, TX 77030 USA; 6 Department of Psychiatry and Behavioral Sciences, McGovern School of Medicine, University of Texas Health Science Center at Houston, Houston, TX 77054 USA; 7 Laboratory of Neurosciences, Graduate Program in Health Sciences, Health Sciences Unit, University of Southern Santa Catarina, Criciúma, SC Brazil

**Keywords:** Neuroscience, Physiology

## Abstract

Defective aquaporin4 (AQP4)-mediated glymphatic drainage has been linked to tauopathy and amyloid plaque in Alzheimer’s disease. We now show that brain interleukin33 (IL33) is required for regulation of AQP4 expression in astrocytes, especially those at neuron-facing membrane domain (n-AQP4). First, IL33-deficient (*Il33*^*−/−*^) mice showed a loss of n-AQP4 after middle age, which coincided with a rapid accumulation of abnormal tau in neurons and a reduction in drainage of abnormal tau to peripheral tissues. Second, injection of recombinant IL33 induced robust expression of AQP4 at perivascular endfoot (p-AQP4) of astrocytes, but not n-AQP4, in *Il33*^*−/−*^ brains. Although the increased p-AQP4 greatly accelerated drainage of intracerebroventricularly injected peptides, it did not substantially accelerate drainage of abnormal tau. These results suggest that p-AQP4 drives overall convective flow toward perivenous space, i.e., glymphatics, whereas n-AQP4 may generate an aqueous flow away from neurons to remove neuronal wastes, e.g., abnormal tau. We have previously shown the role of brain IL33 in DNA repair and autophagy in neurons with oxidative stress. Now, we show that IL33 deficiency also impairs glymphatic drainage. Defects in those mechanisms together may lead to chronic neurodegeneration and tauopathy at old age in IL33-deficient mice.

## Introduction

Sporadic Alzheimer’s disease (AD) and related dementia are an increasing socioeconomic burden [1]. Mounting evidence suggests that ineffective clearance of neuronal wastes such as abnormal tau and amyloid β contributes to pathogenesis of these diseases [2–6]. Glymphatic system drains brain wastes [7]. Its deficiency has been linked to amyloid β plaques and tau deposition [8]. Aquoporin4 (AQP4), a bidirectional water channel, is mainly expressed in astrocytes and ventricular ependymocytes in the brains [9–11]. Astrocyte AQP4, which constitutes a critical part of glymphatic system, locates in either perivascular endfoot (p-AQP4) or neuron-facing membrane domains (n-AQP4) of astrocytes [9, 12]. In addition to regulation of ion exchange and permeability, astrocyte AQP4 also creates water mobilization to promote convective flow toward perivenous space to bring neuronal wastes to glymphatic system [10–13]. These wastes include abnormal tau, e.g., phospho-tau (Ser200/Thu205), and paired helical fragment (PHF1). However, it remains unclear how AQP4 expression in astrocyte is regulated. This study explores whether a cytokine interleukin33 (IL33) is involved in regulation of AQP4 expression in astrocytes.

IL33 is a member of the interleukin1 cytokine family, and has multiple functions in immune defense [14]. Constitutive expression of IL33 in a wide range of tissues, however, suggests its potential role in tissue homeostasis [15]. We recently discovered a critical role of IL33 in eliminating degenerating tissue [16, 17]. Recent studies also hint IL33’s role in brain tissue homeostasis as well. IL33 may be required for injury healing in central nervous system (CNS) [18, 19]. IL33 expression in astrocytes increases with age in mice [20]. IL33 is genetically linked to human AD [21]. Recombinant IL33 (rIL33) ameliorates AD-like symptoms in a human mutant APP transgenic (Tg) mice [22].

Our previous study in IL33-deficient mice (*Il33*^*−/−*^) revealed a critical role of IL33 in rejuvenation of stressed neurons. We first detected an abrupt surge of oxidative stress in the cerebral cortical and hippocampal neurons at their midlife, which generates massive damaged biomolecules such as DNA double-strand breaks (DSB) [20]. IL33 upregulates two mechanisms, i.e., repair of DSBs and autophagic digestion of damaged molecules, to counter the oxidative stress in neurons. It is worthwhile to mention that defects in these two mechanisms have been linked to AD pathogenesis. *Il33*^*−/−*^ mice develop chronic neurodegeneration in those brain regions and AD-like dementia at late life. Old *Il33*^*−/−*^ mice also show accumulation of insoluble tau, e.g., PHF1 in their cortex and hippocampus, which is a hallmark for AD. So far as we know, *Il33*^*−/−*^ model probably was among few to show tau abnormality in neurons beyond mutant human MAPT Tg mice. This finding is highly relevant to human sporadic AD tauopathy, as it is not related to mutant tau genes. As defective glymphatic drainage has been linked to amyloid plaque and tauopathy, we asked if IL33 was also involved in the regulation of glymphatic drainage. The present study reveals that brain IL33 is also critical for glymphatic drainage, probably by regulating AQP4 expression in astrocytes especially after neuronal oxidative surge at middle age.

## Materials and methods

### Mice and their treatment

C57BL/6 (B6) mice were purchased from Harlan (Indianapolis, IN, USA). *Il33*^tm1(KOMP)Vlcg^ (*Il33*^*−/−*^) mouse strain was created through the KOMP Repository (WWW.KOMP.org) and the Mouse Biology Program (www.mousebiology.org) at the University of California Davis [23]. This *Il33*^*−/−*^ strain has been characterized and showed generally normal without any developmental defects [16]. *Il33*^*−/−*^ mice develop aging-related neurodegeneration [20]. All animal procedures in this study were approved by institutional animal welfare committee. For tissue sampling, mice were perfused with room temperature phosphate-buffered saline (PBS) followed 2% paraformaldehyde, and their brains were harvested. In some cases, brains were harvested without fixation, and directly used for isolation of nuclei, organelles, protein, or total RNA with a kit from Ambion (Austin TX, US). A small portion of the brains was snap-frozen in liquid nitrogen. In some cases, kidneys were also harvested for histology or other purposes. Unless specifically stated, five female mice, which were randomly selected from >10, were used for an experimental or control group. These sample sizes were estimated based on previous experiences.

### In vivo treatment of mice with rIL33

A published method was followed with modifications [22]. Murine rIL33, which contains cytokine domain of IL33, was purchased from eBiosciences (Waltham, MA USA). Each of five *Il33*^*−/−*^ mice at week 65 was injected i.p. with 100 μl rIL33 (500 ng/ml) at day 0; this dose was equivalent to 2 μg per 1 kg bodyweight. Mice received the same dose of rIL33 again at day 7, and killed at day 17. As controls, other five littermate *Il33*^*−/−*^ mice were injected with PBS alone. Brains and kidneys were harvested for various purposes. Intracerebroventricular injection (ICV) of a marker peptide was carried out following a published method in treated mice [24]. Five μl of 10 mM pCol (an irrelevant 13-mer peptide) per mouse was injected [25]. The animals were killed 24 h late, and their blood and organs (brains, kidneys, liver, and selected lymph nodes) were harvested for various purpose. Sera were used for measuring pCol peptide by enzyme-linked immunosorbent assay (ELISA) using a special rat antibody to pCol in order to minimize the background [25].

### Histology, immunofluorescence, and immunohistochemistry

Brain tissues, fixed through perfusion, were embedded in paraffin and used for routine histology, including hematoxylin–eosin staining and Bielschowsky silver staining. For Bielschowsky silver staining, 30 μm sections were used. Brain tissues, fixed or non-fixed depending on activity of the antibodies to be used, were frozen and 3 μm frozen sections were cut. All sections were blocked in 3% bovine serum albumin with CD16/32 antibodies. If biotin-labeled antibodies were to be used, a biotin and avidin blocking step were added (Vector BioLab, Philadelphia, PA, USA). Up to four colors, i.e., green (fluorescein isothiocyanate (FITC) or Alex488) red (TRITC, PE, Alex594/555), false purple (APC or Alex647), and blue (DAPI for nuclei as counter-staining) were applied for each section. The tissue sections observed by a confocal microscope (Nikon C2^+^ Eclipse T*i*), or a fluorescent microscope (Nikon 80i Eclipse) and digital images were captured. All digital images were analyzed with NIS Elements 3.2 from Nikon for fluorescent intensity, cellular area, or cell numbers. For quantitation of AQP4 expression, digital imagines from ten randomly selected fields from each of five mice were captured; regions of interest (ROI expressed by pixels), or ROI with fluorescent density, which were eventually calculated into integrated optical density (IOD) per field (1048^2^ pixels), as AQP4 expression level. In some cases, n-AQP was identified and quantitated by the size and colocalization with tubulin β3. For quantitation of PHF1, 30 cortical neurons, or 15 glomeruli from five imagines of the same animal were randomly selected, and IOD for each neuron or glomerulus measured. The results for each mouse of the same group was first compared with others for similar distribution before merging into one group. Tissue samplings and staining, all the above measurements, and final statistical analyses were performed separately by different individuals. For immunohistochemistry, secondary reagents (avidin-peroxidase or peroxidase-conjugated secondary antibody) were used to generate a brown color deposition on tissue section using 3,3' diaminobenzidine as a substrate in the presence of H_2_O_2_. The slides were counter-stained by hematoxylin. A small portion of renal tissues was processed for transmission EM [20].

### Antibodies

Following antibodies were used in this study: biotin goat anti-mouse IL33 (R&D System, Minneapolis, MN, USA), monoclonal rat anti-mouse IL33 (clone 396118, ProSci, Charleston, SC, USA), rabbit anti-glial fibrillary acidic protein (GFAP) (astrocyte marker, Sigma-Aldrich, St. Louis, MO, USA), mouse monoclonal anti-tubulin β3 (neurons, R&D system), mouse monoclonal antibody AT8, which recognizes phosphor-tau(Ser202/Thr205) (ThermoFisher, Waltham, MA, USA), mouse monoclonal antibodies to PHF1 tau (Sigma-Aldrich), FITC-labeled or non-labeled anti-α-actin (Sigma-Aldrich), and rabbit anti-AQP4 antibody (Millipore). Secondary reagents Alexa-555, Alexa-594, and Alexa-647-labeled (Life Technologies, Carlsbad, CA, USA) and phycoerythrin-labeled (BD Biosciences) streptavidin were used to visualize biotin-labeled antibodies. Biotin/avidin and anti-mouse CD16/32 monoclonal antibodies (D34-485, BD Biosciences) were used for blocking non-specific IgG binding. Various immunoglobulin isotypes used as negative controls were from BD Biosciences.

### Western blot

Various proteins, include crude homogenate of cortex and isolated glomeruli were quantitated (Epoch Gen5, BioTek, Winooski, VT), and mixed at 1:1 with SDS sample buffer following our published method [20]. The membrane was incubated with IRDye®800CW labeled secondary antibody for target protein and IRDye® 680LT anti-mouse IgG antibody (LI-COR, Lincoln, NE). The membrane was simultaneously scanned at both wave lengths on an infrared fluorescence scanner (Odyssey, LI-COR), with target protein as green and control α-actin as red. Each band was digitally quantitated as IOD.

### Statistics

Statistical analyses were performed with Prism GraphPad version 3.03 software. Unpaired *t* tests were used for comparison between two groups. For three groups, one-way analysis of variance was performed. Before pooling data from multiple individuals, data from each were statistically compared to rule out any differences among them. Linear regression test was used for analysis of correlations between two groups, e.g., cortical PHF1 vs glomerular PHF1; *r*^*2*^ and *P* value for deviation from zero was calculated for each progression. In addition, frequency distribution was used for frequency distribution of AQP4^+^ staining. Statistical significances were indicated by *(*p* < 0.05), **(*p* < 0.01), or ***(*p* < 0.001).

## Results

### AQP4 expression in astrocytes during neuronal oxidative surge in mice at middle age

**Fig. 1:**
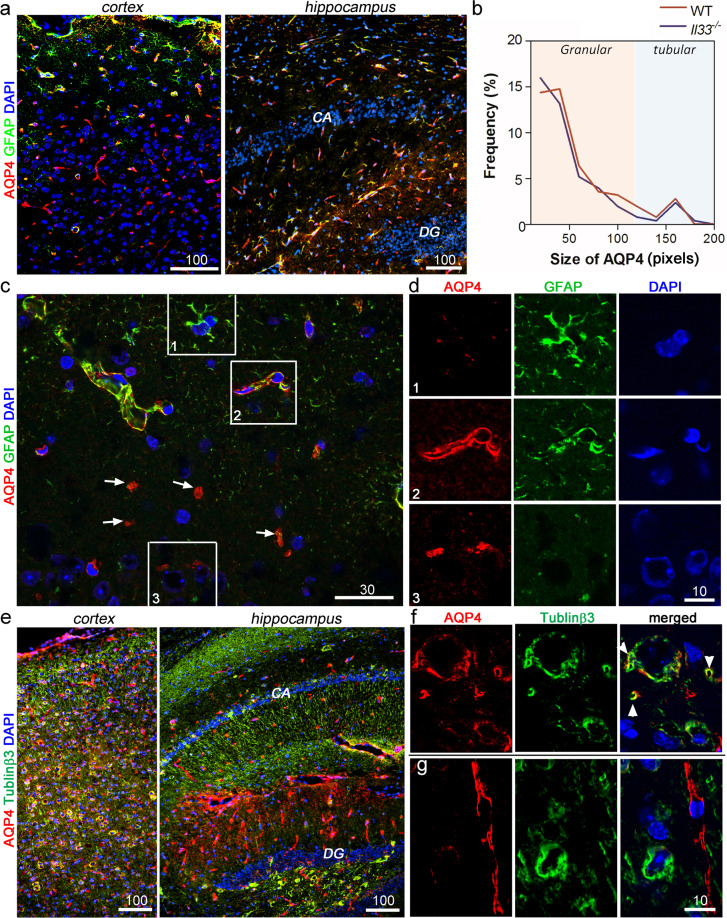
Aquaporin4 (AQP4) expression patterns in mouse brains at 30 weeks. **a** Immunofluorescence shows various co-expression pattern of AQP4 (red) with GFAP^+^ astrocyte (green) in the cortex and hippocampus. **b** Size frequency distribution for each AQP4^+^ region in the cortex reveals two distinguishable sizes of AQP4^+^ regions (i.e., large tubular/linear and small granular). **c** Immunofluorescence shows co-expression of GFAP and tubular/linear AQP4s in perivascular areas (p-AQP4); in contrast, majority granular AQP4s (arrows) do not colocalize to GFAP. **d** Enlarged numbered boxes in **c** show each fluorescent channel to reveal colocalization pattern; box 1, GFAP^+^ astrocyte without AQP4, Box 2, GFAP^+^ astrocytes with tubular/linear p-AQP4; box 3, granular n-APQ4^+^ does not colocalized to GFAP in proximity of neurons identified by nuclear morphology (DAPI). **e** Immunofluorescence shows colocalization patterns of granular n-AQP4 (red) and tubulinβ3 (green) in the cerebral cortex and hippocampus. *CA*, cornu ammonis; *DG*, dentate gyrus. **f** and **g** Representative areas at high magnification in the cortex and hippocampus show each fluorescent channel to reveal colocalization of granular n-AQP4 with neurons or neurites (green, arrowheads in **f**), indicating n-AQP4 in neuron-facing membrane of astrocytes; p-AQP4 is not colocalized to tubulinβ3. Bars’ unit = μm.

As described above, AQP4 distribution in astrocytes is polarized to either p-AQP4 or n-AQP4 [9, 12]. We focused on AQP4 expression at two cellular locations of astrocytes before, at, or after neuronal oxidative stress surge at middle age (45 weeks) [20]. Following many previous studies, we first established AQP4 staining patterns to distinguish p-AQP from n-AQP4 by multi-color immunofluorescence, i.e., combination of AQP4, GFAP as astrocyte marker, and tubulinβ3for neurons. We first examined AQP4 at 30 weeks (i.e., before the oxidative surge). Similar to studies by other groups, two distinct staining patterns for AQP4 were found in the cortex and hippocampus (Fig. 1a), i.e., a large tubular or linear p-AQP4 and a small granular n-AQP4. Two distinct staining patterns were also revealed by size-based frequency distribution analysis (Fig. 1b). Tubular/linear p-AQP4s, which were along astrocyte’s perivascular endfeet, were easily recognizable as they outlined micro-capillaries (Fig. 1c, d box 2), which were never colocalized to neuron-specific tubulinβ3 (Fig. 1e, g). Smaller granular n-AQP4 was more numerous, which were often colocalized with tubulinβ3 but not GFAP (Fig. 1d box 3, Fig. 1f). Thus, these granular n-AQP4s were on the neuron/neurite-facing membrane domains of astrocytes. In the cortex, the majority of p-AQP4 was associated with GFAP^-^ astrocytes (Fig. 1a). In contrast, over 60% of p-AQP4 in hippocampus was colocalized with GFAP^+^ astrocytes (Fig. 1a). n-AQP4 was much fewer in hippocampus, and was still associated with tubulinβ3. CA and dentate gyrus had much less AQP4 (Fig. 1e). Thus, p- and n-AQP4 in astrocytes could be quantitated on the brain tissue sections by their size/shape and by their colocalization with tubulinβ3 after immunofluorescence.

**Fig. 2:**
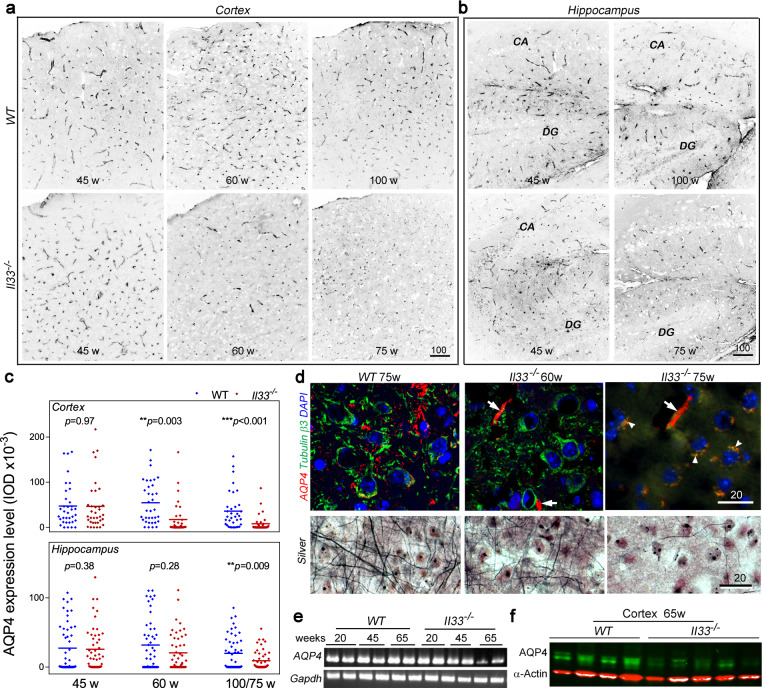
Brain AQP4 expression patterns in *WT* and *Il33*^*−/−*^ mice at or after neuronal oxidative stress surge at their middle age (40–45 weeks). **a**, **b** Micrographs are reverted monochrome for FITC channel of AQP4 staining in the cerebral cortex **a** or hippocampus **b** in *WT* or *Il33*^*−/−*^ mice, revealing significant reduction of AQP4 after 60 weeks in *Il33*^*−/−*^ brains. *CA*, cornu ammonis; *DG*, dentate gyrus. **c** Statistical summary for immunofluorescence-based detection of AQP4 expression level demonstrates a significant reduction of AQP4 in both cerebral cortex and hippocampus at/after 60 weeks as compared with *WT* mice. *IOD*, integrated optical density. **d** Immunofluorescence shows disappearance of n-AQP4 in *Il33*^*−/−*^ brain at or after 60 weeks; note the presence of tubular/linear p-APQ4 (arrows) in *Il33*^*−/−*^ brains at 60 and 75 weeks; numerous age-related pigments in 75-week *Il33*^*−/−*^ brain are indicated by arrowheads. Silver staining (lower panels) demonstrates substantial numbers of neurites in *Il33*^*−/−*^ brain at 60 weeks, and nearly none at 75 weeks. **e** RT-PCR on cortical RNA shows a reduction of *Aqp4* mRNA in *Il33*^*−/−*^ brains after 45 weeks. **f** Western blot of the cortical proteins shows reduction of AQP4 (green) in *Il33*^*−/−*^ mice at 65 weeks; α-actin (red) was used as internal control and probed simultaneously; its staining saturation is revealed (white) for more accurate evaluation. Bars’ unit = μm.

We next examined AQP4 expression at (45 weeks) or after (60–100 weeks) the neuronal oxidative stress surge. In the cortex, p-AQP4 remained nearly unchanged, and then reduced between 60 and 100 weeks (Fig. 2a). However, n-AQP4 showed a brief but significant increase between 45 and 60 weeks, followed by a gradual reduction from 60 and 100 weeks (Fig. 2a). A similar but much less-significant change in AQP4 expression in hippocampus was also observed (Fig. 2b). Statistical analyses on quantitated AQP4 in both cortex and hippocampus by average IOD/unit area confirmed the above results (Fig. 2c). RT-qPCR detected a 1.3-fold increase in *Aqp4* mRNA in the cortex between 45 and 65 weeks (Fig. 2e). In summary, AQP4 expression in astrocytes, especially in their neuron-facing membranes, i.e., n-AQP4, increased after 45 weeks, which was co-incident with the neuronal oxidative stress surge.

### Substantial reduction of n-AQP4 expression in *Il33*^*−/−*^ mice after middle age

We have previously reported that *Il33*^*−/−*^ mice develop accumulation of abnormal phospho-tau(Ser202/Thr205), PHF1 and insoluble tau in neurons, accompanied with neurodegeneration in the cortex/hippocampus after 70 weeks [20]. We next asked if IL33 deficiency also affected AQP4 expression and glymphatic system. *Il33*^*−/−*^ brains at or before 45 weeks (i.e. before the oxidative stress surge) showed a similar expression pattern of AQP4 in both the cortex and hippocampus to age-matched *WT* mice (Fig. 1b, Fig. 2a–c). However, *Il33*^*−/−*^ mice showed a significant reduction in AQP4 expression at 60 weeks, in contrast to an increase in *WT* mice (Fig. 2a, c). At 75 weeks, cortical AQP4 in *Il33*^*−/−*^ mice reduced to only ¼ of much older 100-week-old *WT* mice (Fig. 2a, c). Detailed analysis revealed that the reduction in AQP4 in *Il33*^*−/−*^ brains was not even. Granular n-APQ4 significantly reduced at 60 weeks; there nearly had no n-AQP4 at 75 weeks despite of presence of tubular/linear p-AQP4 (Fig. 2d). We have previously shown neuron/neurite loss after 65–70 weeks in *Il33*^*−/−*^ mice [20]. We asked if reduction of n-AQP4 was due to neuron/neurite loss. Co-staining of AQP4 with tubulinβ3 or silver staining showed that reduction/disappearance of n-AQP4 at 60 weeks preceded neurons/neurites loss after 70 weeks (Fig. 2d, Figure S1). Thus, disappearance of n-AQP4 was not a consequence of neuron/neurite loss. Reduced AQP4 expression in hippocampus was also observed. As compared to *WT* mice, *Il33*^*−/−*^ hippocampus showed no significant reduction in tubular/linear p-AQP4 but significant reduction in n-AQP4. There was a 1.9-fold decrease in total AQP4 at 75 weeks as compared with *WT* mice (Fig. 2c). RT-PCR showed a significant reduction of *Aqp4* mRNA in the *Il33*^*−/−*^ cortex (Fig. 2e). Western blot on cortical proteins confirmed the reduction in cortical AQP4 proteins (Fig. 2f).

We next asked whether AQP4 expression in *Il33*^*−/−*^ mice generally reduces in any location owing to IL33 deficiency. AQP4 is constantly expressed in renal distant tubules and collecting tubes in mice [26]. Immunofluorescence did not detect any differences between *WT* and *IL33*^*−/−*^ mice in terms of both distribution pattern and intensity of AQP4 at 75 weeks (Figure S2a, b). Western blot also showed a similar quantity of AQP4 with similar staining patterns between *Il33*^*−/−*^ and *WT* mice (Figure S2c). Histology did not reveal any abnormality in renal tissue of *Il33*^*−/−*^ mice at 75 weeks (Figure S2d). Thus, reduction of n-AQP4 in astrocytes after 45 weeks was tissue specific. It suggests that IL33 is critical for expression of astrocyte AQP4 especially n-AQP4, but not for AQP4 expression in other tissues.

### Decrease of AQP4 in astrocytes in aged *Il33*^*−/−*^ mice correlates with reduction in abnormal tau drained to glomerular mesangial cells

**Fig. 3:**
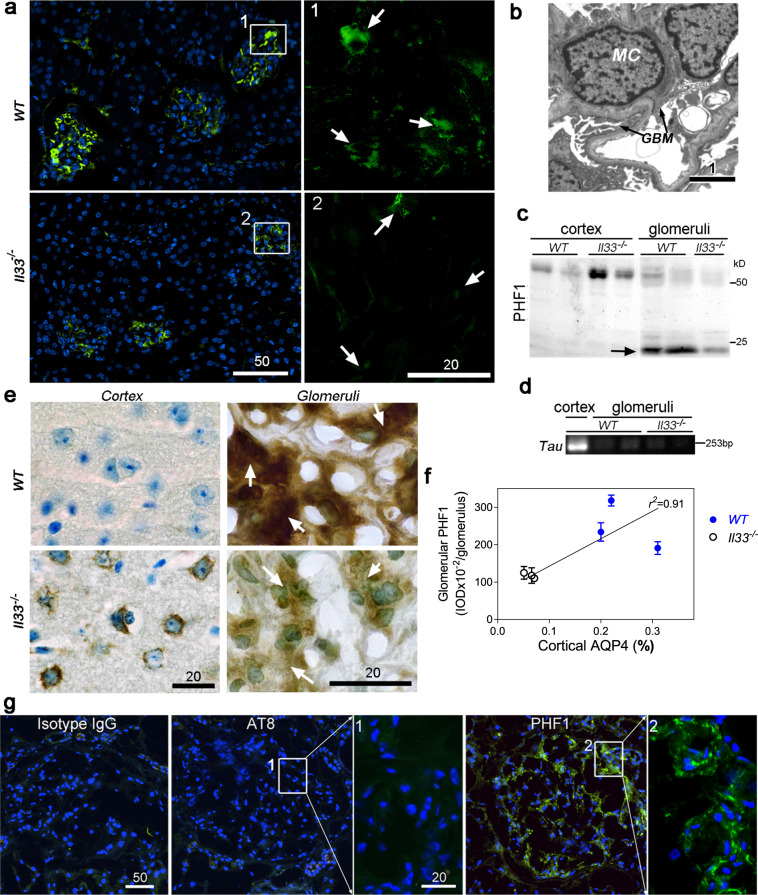
Rapid accumulation of PHF1 tau in *Il33*^*−/−*^ brain after oxidative stress surge positively correlates to reduction of PHF1 tau in mesangial cells in their kidneys. **a** Immunofluorescence shows more PHF1 (green) in glomeruli of *WT* mice than *Il33*^*−/−*^ littermates at 70 weeks; right panels are enlarged boxed areas of left panels to show PHF1 in cytoplasm of glomerular mesangial cells (arrows). **b** Electron microscopy shows structural relationship of mesangial cells (MC) with other cells and glomerular basement membrane (GBM). **c** Western blot shows PHF1 in the cerebral cortex and isolated glomeruli of *WT* or *Il33*^*−/−*^ mice at 70 weeks; note an additional band of low molecular weight of PHF1 (arrow) among glomerular proteins, which is absent in cortical proteins. **d** RT-PCR shows a lack of tau mRNA in glomeruli as compared to the cortex in both *WT* and *Il33*^*−/−*^ mice. **e** Immunohistochemistry on PHF1 in the cerebral cortical and glomerular tissue from the same representative *WT* or *Il33*^*−/−*^ mice reveals a reverse correlation between PHF1 in the two tissue locations, i.e., more neuronal PHF1 vs less glomerular PHF1 (arrows) in *Il33*^*−/−*^ mice, and vice versa in *WT* mice. **f** Statistical analysis shows a positive regression between glomerular PHF1 and cortical AQP4 among *WT* and *Il33*^*−/−*^ mice. *IOD*, integrated optical density. **g** Immunofluorescence on normal human kidneys reveals the presence of PHF1, but not phospho-tau(Ser202/Thr205) (AT8), in glomeruli; an isotype IgG control is also shown; numbered boxed areas are enlarged to show PHF1 in mesangial cells. Bars’ unit = μm.

AQP4-generated convective flow is critical for glymphatic drainage. Recent studies have shown a constant drainage of abnormal tau or amyloid β from brains through glymphatic system [27]. Defective glymphatic system, therefore, has been implicated in plaque formation and tau deposition [8, 10, 27]. Increased AQP4 expression in astrocytes after the neuronal oxidative stress surge prompted us to examine changes in glymphatic drainage. We have previously reported absence of abnormal tau, i.e., phospho-tau(Ser202/Thr205) (by AT8 antibody), PHF1, or pathological tau conformation (by MC1 antibody) in brain sections of *WT* mice in all ages [20]. However, western blot did detect a trace amount of phospho-tau(Ser202/Thr205) and PHF1 in normal cortex after 65 weeks (Fig. 3c), suggesting a balance between generation and drainage of abnormal tau in old *WT* brains. In fact, previous studies have shown the drainage of abnormal tau to peripheral blood and tissues such as livers and kidneys [28]. We further asked which organs or tissues eliminated drained abnormal tau in the blood. Because kidneys are the major excretory organs to remove the internal metabolic wastes, we examined renal tissues from old mice (65 weeks). With focus on phospho-tau(Ser202/Thr205) and PHF1, immunostaining detected a significant amount of hydrophobic PHF1, but not hydrophilic phospho-tau(Ser202/Thr205) in renal glomeruli at 65 weeks (Fig. 3a). In contrast, PHF1 was un-detectable in other organs (i.e., livers, lungs, and lymph nodes). Detailed analysis demonstrated that PHF1 was in cytoplasm of renal mesangial cells, which are closely associated with glomerular basement membrane (GBM) (Fig. 3a, b). We next analyzed proteins from isolated glomeruli. Western blot analysis revealed two bands of PHF1 among glomerular proteins: a minor one at 55kD, which was similar to PHF1 in the cortex, and a major one of low MW (<25 kD), which was absent in cortical proteins (Fig. 3c). This low MW band may represent degraded PHF1. Tau may be transiently expressed by renal podocytes in response to injury of their foot processes [29, 30]. We asked whether the detected glomerular PHF1 was locally expressed by podocytes. The following results and factors demonstrated that PHF1 was not locally expressed in the glomeruli. First, PFH1 was NOT located in podocytes, but rather mesangial cells (Fig. 3b). Second, although a trace amount of normal tau has been detected in the injured kidney, abnormal PFH1 tau in kidneys has never been detected. Third, using cortical mRNA as a control, RT-PCR did not detect any tau mRNA in glomeruli of *WT* or *Il33*^*−/−*^ mice (Fig. 3d). Fourth, mesangial cells are well known to capture and digest insoluble macromolecules trapped by GBM. Fifth, majority of PHF1 in mesangial cells were fragments of small molecules, which were not found in the brains (Fig. 3c). Thus, PHF1 and their fragments in mesangial cells were most likely from the brains, which may be a reliable index for drainage of insoluble tau from brains.

We asked if reduced n-AQP4 in *Il33*^*−/−*^ brains had weakened glymphatic drainage of abnormal tau, which would lead to less glomerular PHF1. Both immunofluorescence and western blot showed much less PHF1 in glomeruli of *Il33*^*−/−*^ mice as compared with age-matched *WT* mice at 65 weeks (Fig. 3a, c). Comparison of immunostainings for PHF1 tau between the brains and kidneys from the same individuals showed a reverse correlation, i.e. no cortical PHF1 vs more glomerular PHF1 in *WT* mice, in contrast to much more cortical PHF1 vs less glomerular PHF1 in *Il33*^*−/−*^ mice (Fig. 3e). Statistics revealed a positive regression between glomerular PHF1 and cortical AQP4 among *WT* and *Il33*^*−/−*^ mice (Fig. 3f). These suggested the involvement of AQP4, probably n-AQP4, in draining PHF1 to glomeruli.

We next asked whether human kidneys also contain abnormal tau. Interestingly, we were able to detect hydrophobic PHF1 tau, but not phospho-tau(Ser202/Thr205) in mesangial cells by immunofluorescence in all three normal human kidneys (Fig. 3g).

### Injection of rIL33 enhances both p-AQP4 expression and drainage of brain ventricular fluid in aged *Il33*^*−/−*^ mice

**Fig. 4:**
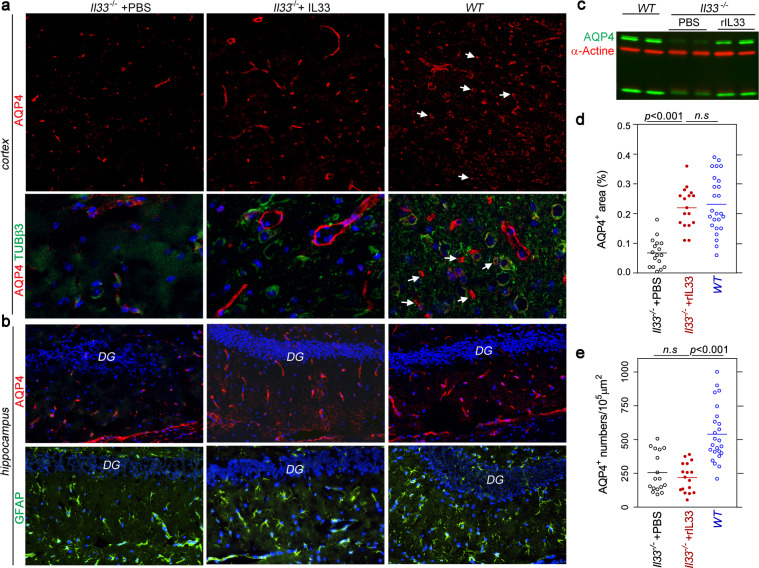
Injection of recombinant IL33 induces expression of a high level of p-AQP4 in astrocytes in aged *Il33*^*−/−*^ brains. **a** Immunofluorescence shows a significant increase in p-AQP4 (red) expression in the cortex of *Il33*^*−/−*^ mice after receiving rIL33 injection (*Il33*^*−/−*^+rIL33) as compared with PBS controls (*Il33*^*−/−*^+PBS); two-color staining (lower panels) reveals high density of p-AQP4 (red) but nearly no granular n-APQ4 around neurons (green, tubulinβ3) in *Il33*^*−/−*^+rIL33 mice, in contrast to many (arrows) in *WT* mice. Also note much less tubulinβ3 in both *Il33*^*−/−*^+rIL33 and *Il33*^*−/−*^+PBS mice than *WT* mice. **b** Immunofluorescence shows an increase in p-AQP4 expression in hippocampus (upper panels) of *Il33*^*−/−*^+rIL33 mice; note that GFAP^+^ astrocyte (green) population is similar among three groups (lower panel). **c** Western blot on cortical proteins shows quantity of AQP4 in *Il33*^*−/−*^+rIL33 mice is comparable to *WT* mice, and much higher than *Il33*^*−/−*^+PBS controls. **d**, **e** Statistics on total AQP4 expression (% of area) (**d**), and numbers of AQP4^+^ regions (**e**) in the cortex demonstrates that rIL33 injection enhances total AQP4 expression level but not numbers of AQP4^+^ regions.

We asked whether exogenous IL33 could enhance APQ4 expression in astrocytes, and thus, accelerate glymphatic drainage in aged *Il33*^*−/−*^ mice. We have previously shown that *Il33*^*−/−*^ mice begin to develop accumulation of PHF1 after 65 weeks [20]. Five *Il33*^*−/−*^ mice of 65 weeks received rIL33 twice through intraperitoneal injection at a 7-day interval. Their brains and kidneys were examined for AQP4 expression and PHF1 10 days after the second injection. All rIL33 recipient *Il33*^*−/−*^ (*Il33*^*−/−*^+IL33) mice showed a robust increase in AQP4 expression in the cortex, and a moderate increase in hippocampus, as compare to PBS control (*Il33*^*−/−*^+PBS) (Fig. 4a, b). Quantitatively, the overall AQP4 in the cortex (% of AQP4^+^ area) of *Il33*^*−/−*^+IL33 mice was statistically comparable to that of *WT* mice as revealed by both immunofluorescence and western blots (Fig. 4c, d). However, the increased AQP4 was restricted to perivascular areas (i.e., tubular/linear p-AQP4), while granular n-AQP4 in neuron-facing membrane remained no increase (Fig. 4a). Statistical analysis reflected a similar tendency: despite comparable levels of overall AQP4 for *Il33*^*−/−*^+IL33 and *WT* mice, the number of AQP4 positive regions in *Il33*^*−/−*^+IL33 unchanged (i.e., statistically comparable to *Il33*^*−/−*^ mice) (Fig. 4d, e). Thus, injected rIL33 greatly promoted p-AQP4 expression of astrocytes, but no effect on n-AQP4 expression. We next asked whether reduction or increase in AQP4 expression was related to the number of astrocytes, rather than rIL33. AQP4 expression was associated with GFAP^+^ astrocytes in hippocampus (Fig. 1a). Immunofluorescence on GFAP in hippocampus did not show significant difference in numbers of astrocytes among *Il33*^*−/−*^+IL33, *Il33*^*−/−*^+PBS, and *WT* mice (Fig. 4b). Thus, injected rIL33 was most likely responsible for upregulation of p-AQP4 expression

**Fig. 5:**
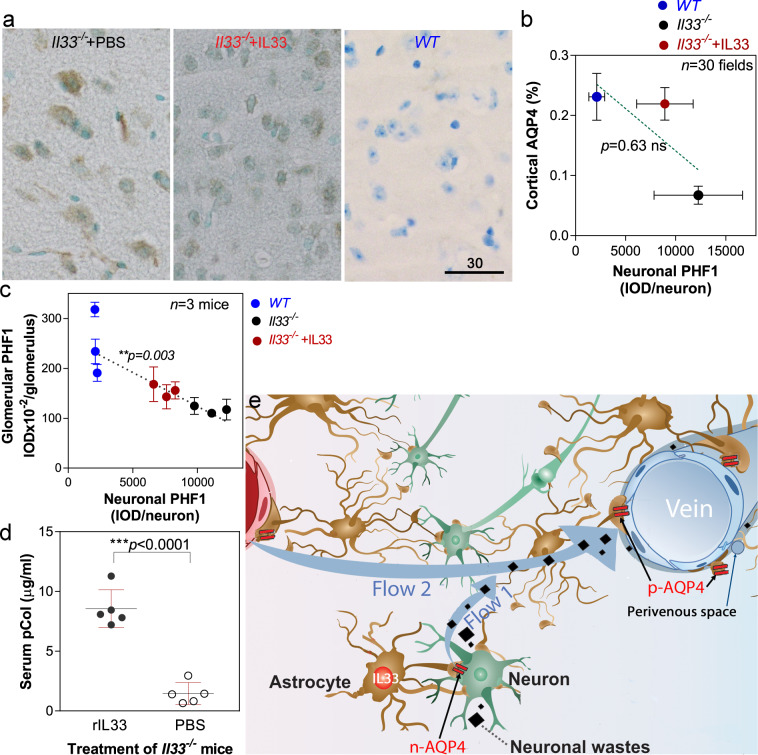
Recombinant IL33 (rIL33) enhances drainage of intracerebroventricularly (ICV) injected peptide, but has limited effect on reduction of neuronal PHF1 tau in *Il33*^*−/−*^ mice. **a** Immunohistochemistry shows neuronal PHF1 in indicated groups; note slightly reduced PHF1 in *Il33*^*−/−*^+rIL33 mice as compared with *Il33*^*−/−*^+PBS controls; no PHF1 was detectable in WT mice. Bars’ unit = μm **b** Regression analysis shows no linear correlation between total cortical AQP4 level (% of area) vs neuronal PHF1 among *Il33*^*−/−*^+PBS, *Il33*^*−/−*^+IL33, and WT mice; note that total AQP4 level in *Il33*^*−/−*^+IL33 mice is not proportional to neuronal PHF1. Neuronal PHF1 was quantitated on digital immunostaining imagines, and expressed as IOD/neurons. **c** Statistical analysis shows a linear negative regression (black dotted line) between cortical and glomerular PHF1 among *WT*, *Il33*^*−/−*^+PBS and *Il33*^*−/−*^+rIL33 mice at 70 weeks; three mice were used for each group. **d** ELISA shows a sixfold increase in serum pCol peptide that had been injected into ventricles 24 h ago in *Il33*^*−/−*^+rIL33 mice, as compared to PBS controls. **e** Diagram depicts two aqueous flows driven by astrocyte AQP4 (lightly colored arrows): Flow 1, driven by n-AQP4, brings neuronal wastes away from neurons, and Flow 2, convective flow driven by p-AQP4 toward perivenous space, flushes brain wastes further to perivenous space, i.e., glymphatics. Therefore, a lack of n-AQP4 results in ineffective removal of neuronal wastes, e.g., PHF1 from neurons.

We examined whether increased p-AQP4 proportionally enhanced drainage of PHF1 tau in *Il33*^*−/−*^+IL33 mice. Immunohistochemistry showed some, but statistically insignificant, reduction in PHF1 in the cortical neurons in *Il33*^*−/−*^+IL33 as compared with *Il33*^*−/−*^+PBS controls (Fig. 5a, b). Immunofluorescence further revealed a small but insignificant increase in glomerular PHF1 in *Il33*^*−/−*^+IL33 group (Fig. 5c). Three groups (*WT*, *Il33*^*−/−*^+IL33 and *Il33*^*−/−*^+IL33) did show a reverse linear correlation between cortical and glomerular PHF1 (Fig. 5c). It further supports that glomerular PHF1 probably was from brains in all three groups, and could be reliable index for drainage of abnormal tau. However, there lacked a lineage regression between cortical PHF1 and cortical AQP4 among the three groups (Fig. 5b). Thus, expected decrease in neuronal PHF1 in *Il33*^*−/−*^ +IL33 mice was not proportional to their increased cortical APQ4 level as compared to *WT* or *Il33*^*−/−*^ +PBS control. It suggests a limited role of p-AQP4 in PHF1 tau drainage from neurons. The above results drove us to examine whether rIL33-induced p-AQP4 was involved in drainage from CNS to peripheral tissues. An irrelevant 13-mer peptide pCol was delivered into the lateral ventricle through ICV injection. The peptide drained to the peripheral blood was measured by ELISA 24 h late. A surprising sixfold increase in drained peptide in sera was observed in *Il33*^*−/−*^+IL33 mice as compared to *Il33*^*−/−*^+PBS control (Fig. 5d). This result revealed a critical role of p-AQP4 in the drainage of brain tissues. On the other hand, very limited improvement in the drainage of neuronal PHF1 tau from brains in rIL33 injected *Il33*^*−/−*^ mice is most likely attributed to the lack of n-AQP4, as rIL33 injection completely failed to induce n-AQP4 expression. Interestingly, rIL33 injection of *WT* mice also induced p-AQP4 expression to a higher level (1.8-fold), and showed an increase in pCol drained to the bloods (Figure S3). However, the increases in both p-AQP4 and pCol drainage were much less significant as seen in *Il33*^*−/−*^ mice.

## Discussion

### Brain IL33 regulates multiple anti-aging mechanisms in aged neurons

Recent studies have revealed that glymphatics drains abnormal tau, amyloid β, and other neuronal wastes from brains, which may prevent AD [10, 11, 27]. AQP4 is vital component of glymphatics. This study showed requirement of brain IL33 in regulation of AQP4 expression in astrocytes, through comparison between *Il33*^*−/−*^ vs *WT* mice at different ages, and between *Il33*^*−/−*^ mice with or without receiving rIL33. We have previously reported an oxidative stress surge in neurons in the cerebral cortex and hippocampus at middle age in mice. To counteract the oxidative stress, IL33 rapidly upregulate the repair of DNA DSBs and autophagic elimination of damaged molecules [20]. This study further showed that brain IL33 was also required for enhancing glymphatic drainage of damaged molecules such as abnormal tau after the neuronal oxidative stress surge by regulating AQP4 expression. In that sense, IL33 is a master regulator for counter-oxidative or anti-aging mechanisms in the stressed neurons after the oxidative stress surge, which include neuronal DNA repair, autophagic, and glymphatic removal of neuronal wastes or damaged molecules. Importantly, defects in these mechanisms are already suspected possible causes for human AD. IL33 deficiency impairs these mechanisms, and subsequently causes intra- or extracellular accumulation of unrepaired damaged molecules or wastes in certain regions of brains. This, in turn, results in chronic neurodegeneration and dementia at late life. Our next question is which pathway does brain IL33 trigger the anti-oxidative response in stressed neurons? Our previously study has reported release of cytokine domain of IL33 in aged brains in mice [20]. Furthermore, expression of IL33’s receptor ST2 has been reported in the cortical and hippocampal neurons in aged normal brains or astrocytes [31]. Those observations suggest that IL33 triggers activation of NF-κB transcription pathway in stressed neurons or astrocytes.

### Astrocyte n-AQP4 (neuron-facing membrane) is critical for drainage of abnormal tau

Our experiments showed that brain IL33 is required for expression of n-AQP4 expression, which is critical for efficient removal of neuronal wastes, e.g., abnormal tau toward glymphatics. Our conclusion is based on the following results. First, IL33 deficiency caused a complete loss of n-AQP4 after middle age. Second, injection of exogenous rIL33 failed to restore expression of n-AQP4. Third, loss of n-AQP4 was associated rapid accumulation of abnormal tau in neurons. It is worthwhile to point out that lack of n-AQP4 in *Il33*^*−/−*^ mice did not cause a significant change in brain water contents since p-AQP4 was much less affected. Thus, *Il33*^*−/−*^ brains seemed “normal” until at their late age. Finally, astrocyte p-AQP4 is known to play a critical role in glymphatic system [10]. Function of n-AQP4, however, remains less clear. Several studies reported that AQP4 in peri-synaptic astrocyte processes may adjust synaptic space and remove neuronal transmitters away from neurons [27, 32–34]. With focus on temporal expression of AQP4, we demonstrated an increase in n-AQP4 when neurons undergo oxidative stress surge after 45 weeks. An increase in n-AQP4 may be one part of counter-oxidative response, which may remove damaged molecules, e.g., PHF tau away from neurons. It is interesting to mention our result that rIL33 induced a high level of p-AQP4 expression and promoted drainage of ICV-injected peptide, but not PHF1. This result seems against n-AQP4’s role in glymphatic drainage. However, this paradox can be nicely explained by the following hypothesis (Fig. 5e). There are two AQP4-driven aqueous flows in the brains: *Flow 1* is generated by n-AQP4 to bring neuronal wastes away from neurons, and *Flow 2* is driven by p-AQP4 to create a convective flow from arteries to perivenous space to further bring these neuronal wastes to glymphatics. In fact, *Flow 2* has been previously well described. Only two flows functioning together can effectively drain neuronal wastes to glymphatics. This hypothesis, in fact, supports the following observed results in our study. First, n-AQP4 positively correlated to PHF1 tau drained to glomeruli, but negatively correlated to neuronal PHF1 among *WT* and *Il33*^*−/−*^ mice, despite similar quantities of p-AQP4 among the mice. Second, rIL33-induced increase in p-AQP4 expression significantly enhanced drainage of ICV-injected peptide to the blood, but much less effective in drainage of neuronal PHF1 tau as n-AQP4 was not upregulated. Finally, a robust reduction of n-AQP4 expression in *Il33*^*−/−*^ brains, but not p-AQP4 at middle age, was co-incident with rapid accumulation of PHF1 in neurons and reduction of glomerular PHF1. A recent study showed that deletion of AQP4 in APP/PS1 mice exacerbates brain Aβ accumulation and memory impairment, suggesting AQP4 involvement in draining amyloid peptides [35]. However, it needs to point out that enhancement of AQP4 expression for accelerating glymphatic drainage is only one mechanism among many others (e.g., DSB repair and autophagic elimination of damaged proteins).

### Exogenous IL33 is less effective in improving drainage of neuronal wastes

A recent paper has shown that injection of rIL33 reduced plaque burden in the brains of human mutant APP Tg, suggesting a potential therapeutic value of IL33 [22]. Our study showed that injected rIL33 induced a high-level expression of p-AQP4, which was accompanied with a robust upsurge of drainage of ICV-injected peptide to the peripheral circulation. On the other hand, drainage of brain PHF1 tau in *Il33*^*−/−*^ mice was not significant improved after rIL33 injection. Furthermore, unlike extracellular amyloid peptides, intracellular abnormal tau would be excluded from neurons by autophagy, exocytosis, and other mechanisms before being removed to glymphatics. Extracellular abnormal tau would be then drained from brains by a tandem two-flow mechanism as described above (Fig. 5e). Injection of exogenous rIL33 induced only p-AQP4 expression, but not n-AQP4, as well other exocytosis mechanisms such as autophagy (unpublished data). As a result, injected rIL33 had a limited effect. Thus, an effective therapeutic rIL33 must be delivered across BBB to mimic endogenous brain IL33 to be more effective.

### Renal mesangial cells are the final destination of drained hydrophobic abnormal tau

Drainage of abnormal tau and amyloid β peptides from aged brains through glymphatic system has been demonstrated [36]. A recent study has shown that cisterna magna injected ^131^I-labeled synthetic tau dynamically effluxed from the brain and was mainly cleared from the kidney, blood, and liver in mice [28]. More-detailed studies further support that changes in the phosphorylation state of tau are temporally associated with metabolic, neurodegenerative, and structural markers of disease [37, 38]. Some phospho-tau, e.g., phospho-tau(217), accompany amyloid β accumulation, whereas others, e.g., phospho-tau(205) become phosphorylated at later stages of the neurodegenerative process. Excessive phosphorylation leads to increased aggregation of tau into insoluble PHF tau, and thereafter into neurofibrillary tangles. Therefore, it is important to address the following questions for elucidation of tauopathy and for identification of biomarkers for early diagnosis: (1) what is the dynamics of drainage of those neuronal wastes into peripheral tissues? (2) does the solubility of a neuronal waste affect glymphatic drainage efficiency and final destinations? Our study may provide some answers to the second question. Drainage of both hydrophobic and hydrophilic neuronal wastes has been reported; these wastes can be detected in both bloods and tissues, e.g., livers. Our study suggested that mesangial cells in glomeruli may be the final destinations of hydrophobic/insoluble tau. Thus, only hydrophobic PHF tau, but not soluble phospho-tau(Ser200/Thr205), was detected in the glomeruli. Importantly, we also observed hydrophobic PHF tau in normal human mesangial cells. Mesangial cells are known to capture and further cleanup macromolecules such as IgA immune complex and DNA trapped by GBM [39, 40]. It is not surprising that mesangial cells also capture and degrade the neuronal wastes, especially those hydrophobic ones, from brains. This finding has potential clinical significance. Several studies have reported detection of amyloid β in urine, which may be used as diagnostic index for AD [41]. On the other hand, interpretation of urinal abnormal tau or amyloid β should be cautious. The quantities of abnormal neuronal proteins alone may not accurately reflect the development of plaque or tauopathy in patients’ brains, because it depends on both their generation rate and glymphatic drainage efficiency. In our model, mesangial PHF1 levels were higher in *WT* mice than *Il33*^*−/−*^ mice. Obviously, it should be interpreted as more efficient glymphatic drainage in *WT*, rather than accumulation of abnormal tau in the brains. In addition, insoluble tau may not be released to urine as they may have been digested in the kidneys.

## Supplementary information

Figrue S1

Figure S2

Figure S3
