# Supplementary figures and images for: Requirement of brain interleukin33 for aquaporin4 expression in astrocytes and glymphatic drainage of abnormal tau
Source: Mol Psychiatry. 2021 Jan 12;26(10):5912–24. doi: 10.1038/s41380-020-00992-0 (PMC8273186; doi:10.1038/s41380-020-00992-0)

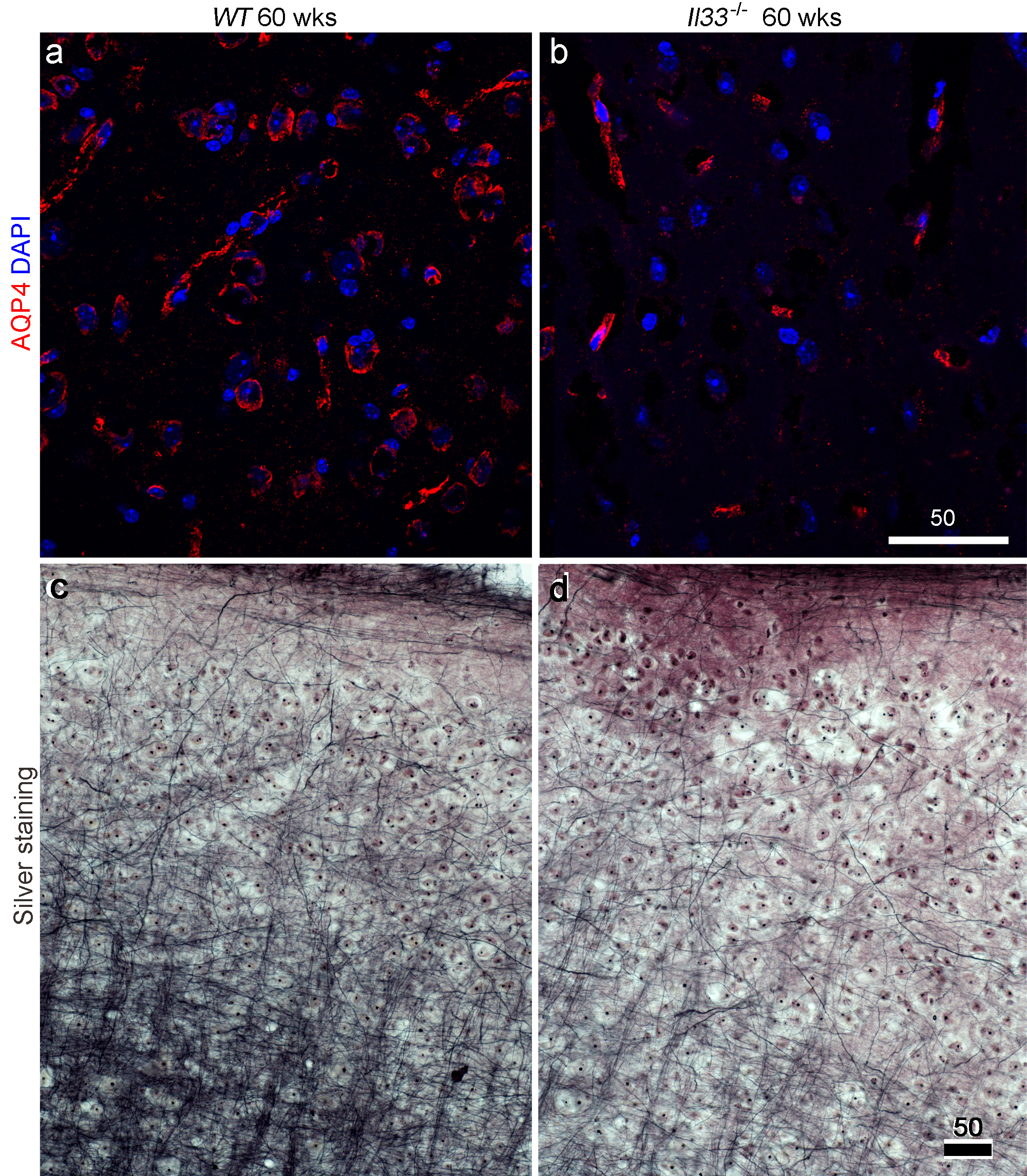

Supplement: Supplementary file 1 — Figrue S1 [file 41380_2020_992_MOESM1_ESM.jpg]

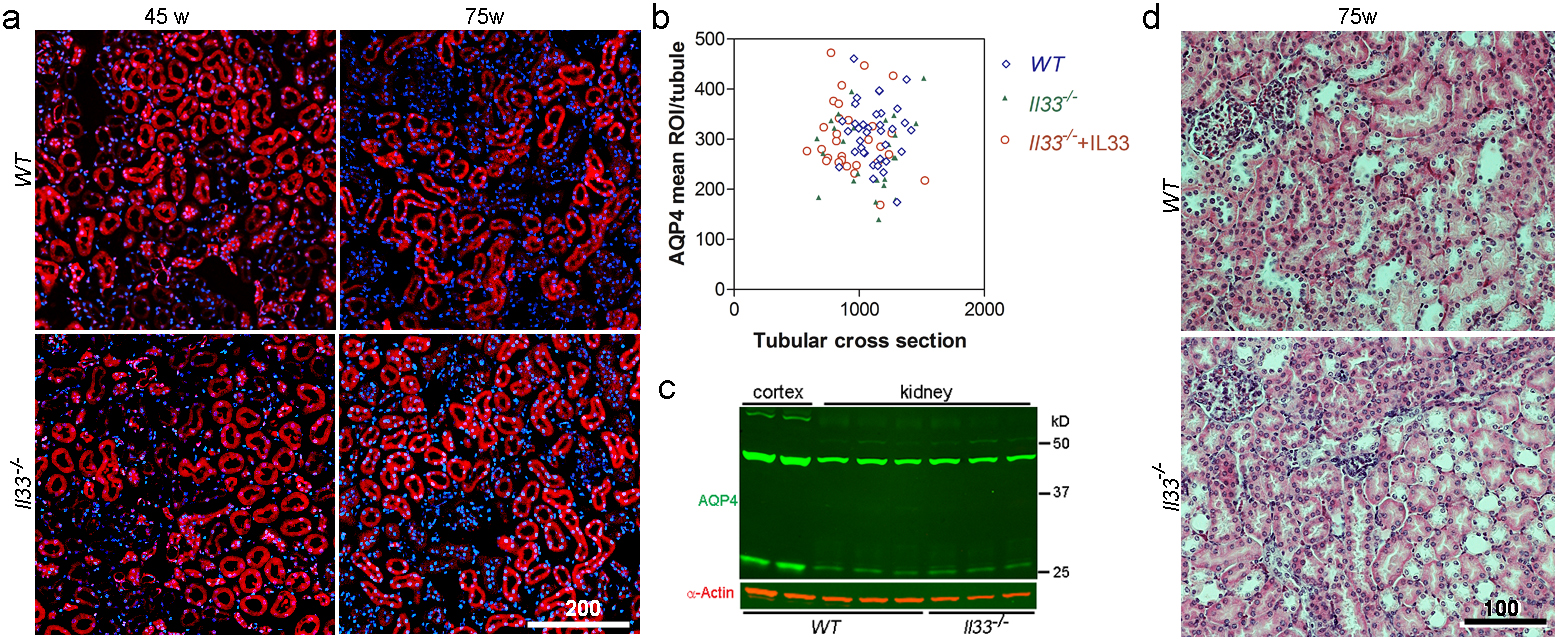

Supplement: Supplementary file 2 — Figure S2 [file 41380_2020_992_MOESM2_ESM.jpg]

**a**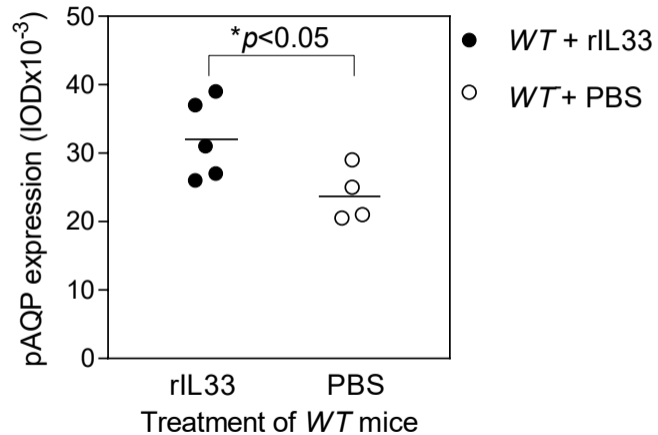**b**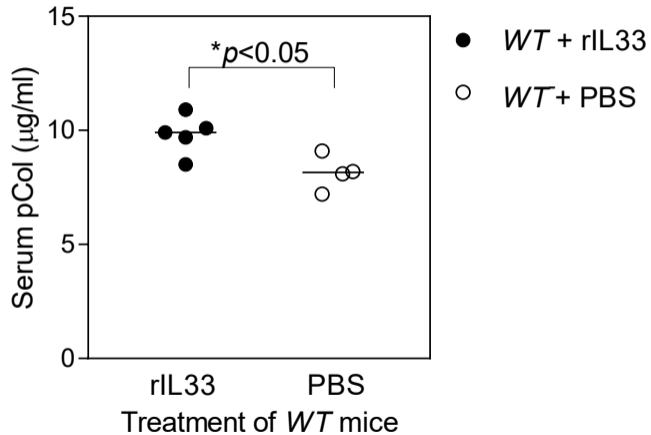

Supplement: Supplementary file 3 — Figure S3 [file 41380_2020_992_MOESM3_ESM.pdf]
